# Ten-Year Outcomes Following Roux-en-Y Gastric Bypass vs Duodenal Switch for High Body Mass Index: A Randomized Clinical Trial

**DOI:** 10.1001/jamanetworkopen.2024.14340

**Published:** 2024-06-03

**Authors:** Odd Bjørn Kjeldaas Salte, Torsten Olbers, Hilde Risstad, Morten Wang Fagerland, Torgeir Thorson Søvik, Ingvild Kristine Blom-Høgestøl, Jon A. Kristinsson, My Engström, Tom Mala

**Affiliations:** 1 Department of Gastrointestinal and Pediatric Surgery, Oslo University Hospital, Oslo, Norway; 2 Center for Morbid Obesity, Department of Endocrinology, Morbid Obesity and Preventive Medicine, Oslo University Hospital, Oslo, Norway; 3 Institute of Clinical Medicine, University of Oslo, Oslo, Norway; 4 Department of Biomedical and Clinical Sciences, Linköping University, Linköping, Sweden; 5 Department of Surgery, Vrinnevi Hospital, Norrköping, Sweden; 6 Department of Endocrinology, Morbid Obesity and Preventive Medicine, Oslo University Hospital, Oslo, Norway; 7 Oslo Centre for Biostatistics & Epidemiology, University of Oslo, Oslo, Norway; 8 Institute of Health and Care Sciences, Sahlgrenska Academy, University of Gothenburg, Gothenburg, Sweden; 9 Department of Surgery, Sahlgrenska University Hospital, Region Vastra Gotaland, Gothenburg, Sweden

## Abstract

**Importance:**

Results from long-term follow-up after biliopancreatic diversion with duodenal switch (DS) are scarce.

**Objective:**

To compare weight loss, health outcomes, and quality of life 10 years or more after Roux-en-Y-gastric bypass (RYGB) and DS surgery in patients with severe obesity—that is, a body mass index (BMI; calculated as weight in kilograms divided by height in meters squared) of 50 to 60.

**Design, Setting, and Participants:**

This open-label randomized clinical trial was conducted at 2 academic bariatric centers in Sweden and Norway. Sixty patients with a BMI of 50 to 60 were included from March 1, 2006, to August 31, 2007. Data were analyzed from August 12, 2022, to January 25, 2023.

**Interventions:**

Laparoscopic RYGB or laparoscopic DS.

**Main Outcomes and Measures:**

The main outcome was change in BMI after 10 or more years. Secondary outcomes included anthropometric measures, lipid and glycemic profiles, bone mass density, adverse events, gastrointestinal tract symptoms, and health-related quality of life.

**Results:**

Forty-eight of the original 60 patients (80%) were assessed after a median of 12 (range, 9-13) years (mean [SD] age, 48.0 [6.0] years; 35 women [73%]). At follow-up, the mean BMI reductions were 11.0 (95% CI, 8.3-13.7) for RYGB and 20.3 (95% CI, 17.6-23.0) for DS, with a mean between-group difference of 9.3 (95% CI, 5.4-13.1; *P* < .001). Total weight loss was 20.0% (95% CI, 15.3%-24.7%) for RYGB and 33.9% (95% CI, 27.8%-40.0%) for DS (*P* = .001). Mean serum lipid levels, except high-density lipoprotein cholesterol and hemoglobin A_1c_, improved more in the DS group during follow-up. Bone mass was reduced for both groups from 5 to 10 years, with lower bone mass after DS at 10 years. Quality-of-life scores (Obesity-Related Problem Scale and the 36-Item Short Form Health Survey) were comparable across groups at 10 years. The total number of adverse events was higher after DS (135 vs 97 for RYGB; *P* = .02). More patients in the DS group developed vitamin deficiencies (21 vs 11 for RYGB; *P* = .008) including 25-hydroxyvitamin D deficiency (19 for DS vs 9 for RYGB; *P* = .005). Four of 29 patients in the DS group (14%) developed severe protein-caloric malnutrition, of whom 3 (10%) underwent revisional surgery.

**Conclusions and Relevance:**

In this randomized clinical trial, BMI reduction was greater after DS, but RYGB had a better risk profile over 10 years. Biliopancreatic diversion with DS may not be a better surgical strategy than RYGB for patients with a BMI of 50 to 60.

**Trial Registration:**

ClinicalTrials.gov Identifier: NCT00327912

## Key Points

**Question:**

What are the outcomes 10 years after Roux-en-Y gastric bypass and duodenal switch surgery regarding weight control, metabolic health, quality of life, and complications in patients with a body mass index (BMI) of 50 to 60?

**Findings:**

In this randomized clinical trial of 60 participants, the reductions in body weight and BMI were greater after duodenal switch. Although metabolic markers improved more after duodenal switch, this procedure was also associated with more adverse effects, including nutritional complications.

**Meaning:**

Duodenal switch may not be superior to Roux-en-Y gastric bypass as treatment for patients with BMI of 50 to 60.

## Teaser

This randomized clinical trial compares 10-year outcomes of Roux-en-Y-gastric bypass and duodenal switch surgery among patients with severe obesity.

## Introduction

Bariatric surgery is the only treatment for severe obesity that has been proven to enable large and sustained weight loss. The weight loss is associated with improvements in metabolic function, reduced incidence of cardiovascular events and certain cancers, improved quality of life, and longer life expectancy.^1,2^ However, bariatric surgery is also associated with long-term adverse effects where the pattern and incidence are not fully understood.

Weight loss and comorbidity resolution may be insufficient after bariatric surgery in patients with severe obesity—that is, a body mass index (BMI; calculated as weight in kilograms divided by height in meters squared) of more than 50. The preferred surgical approach for these patients is debated. Procedures that induce substantial malabsorption, such as biliopancreatic diversion with duodenal switch (DS), are considered by many as an option for patients with very high initial BMI.^3^ However, adverse effects related to malabsorption have limited the use of such methods.^4^

Few controlled studies have evaluated Roux-en-Y gastric bypass (RYGB) compared with DS in patients with a BMI of greater than 50, particularly with follow-up beyond 5 years.^3,5,6,7,8,9,10,11,12,13^ In the ASGARD (Aker and Sahlgrenska Gastric Bypass Randomized vs Duodenal Switch) trial,^14,15^ patients with initial BMI of 50 to 60 were randomized to either RYGB or DS. The primary study end point was a change in BMI 2 years after surgery, while secondary end points included sustainability of weight loss, effects on comorbidity, metabolic and nutritional outcomes, health-related quality of life, and adverse effects. We report outcomes from an extended follow-up of the ASGARD trial participants at 10 years or more after surgery.

## Methods

### Trial Design and Participants

The ASGARD study is a randomized clinical trial performed at Oslo University Hospital, Oslo, Norway, and Sahlgrenska University Hospital, Gothenburg, Sweden. Patients with a BMI of 50 to 60 and aged 20 to 50 years were included between March 1, 2006, and August 31, 2007. The study was approved by ethical committees at both study centers, and all participants gave written informed consent. The study design and results through 5 years of follow-up have been reported previously.^14,15,16^ Follow-up was completed December 13, 2019, and data were analyzed from August 12, 2022, to January 25, 2023. The study was conducted in accordance with the Consolidated Standards of Reporting Trials (CONSORT) reporting guideline. The trial protocol is available in Supplement 1.

### Randomization

Randomization was computer generated using LabVIEW software, version 7 (National Instruments Corp). Participants were stratified by sex, age (<35 or ≥35 years), BMI (<55 or ≥55), and study center.^14,15,16^

### Procedures and Follow-Up

Roux-en-Y gastric bypass and DS were performed laparoscopically. In RYGB, a gastric pouch of less than 25 mL was anastomosed to an antecolic-antegastric alimentary limb of 150 cm with a linear stapler and complementary suturing. The biliopancreatic limb was 50 cm. The 1-stage DS procedure included a sleeve gastrectomy stapled loosely along a 30F to 32F bougie. The duodenum was divided 2 to 3 cm distal to the pylorus. The alimentary limb was 200 cm, the common channel was 100 cm, and the length of the biliopancreatic limb was not measured. Cholecystectomy was not performed during either procedure.

A similar regimen of vitamin and mineral supplementation was prescribed upon hospital discharge and adjusted according to serum concentrations during follow-up.^16^ Patients were scheduled for study visits at 6 to 8 weeks, 6 months, and 1, 2, 5, and 10 years after surgery. From 5 to 10 years after surgery, patients undergoing DS (but not RYGB) were offered routine annual checkups.

### Outcomes

Outcomes were predefined. The primary outcome was change in BMI after 10 or more years. During the consultations, vitamin and mineral levels were evaluated, and supplement regimens were individually adjusted. Dual-energy x-ray absorptiometry scans and levels of serum bone markers (procollagen type 1 N-terminal propeptide [P1NP], a bone formation marker, and type 1 collagen cross-linked C-telopeptide [CTX-1], a bone resorption marker) were measured at 5 and 10 years. Study questionnaires were distributed at baseline and 1, 2, 5, and 10 years after surgery.

### Definitions

Presence and remission of type 2 diabetes was evaluated according to American Diabetes Association recommendations.^17^ Complete remission was defined as fasting glucose level of less than 100 mg/dL (to convert to mmol/L, multiply by 0.0555), and partial remission as fasting glucose level of 100 to 125 mg/dL, both with at least 1 year without antihyperglycemic medication prior to evaluation. Metabolic syndrome was diagnosed according to the International Diabetes Federation.^18^ The hemoglobin A_1c_ (HbA_1c_) measurements changed during the study from percentages to millimoles per mole (both units are included). The standard for measuring low-density lipoprotein cholesterol levels changed from an indirect method (Friedewald formula) to a direct measurement during 2012. For comparative purposes, the indirect method was used. Vitamin deficiencies were defined as blood levels below the laboratory reference values or an increased use of vitamin supplements.^19^ Definitions of comorbidities are presented in eTable 1 in Supplement 2.

### Adverse Events

Information about adverse events and effects, including diagnostic or therapeutic procedures, and hospital admissions were retrieved from the patients and their medical records. Adverse events or effects occurring 30 days after surgery or later are reported. Perioperative complications have previously been described in detail without major differences between the 2 procedures.^15^

### Dual-Energy X-Ray Absorptiometry

Bone densitometry was performed 5 and 10 years after surgery using dual-energy x-ray absorptiometry scanners (Lunar iDXA; GE HealthCare). Mean areal bone mineral density (aBMD; measured in grams per square centimeter) was analyzed for lumbar back (L1-L4), femoral neck, total femur, and total aBMD. Corresponding T and *z* scores were estimated, and osteopenia and osteoporosis were defined based on World Health Organization criteria and International Society for Clinical Densitometry official position statements.^20^ Scans were evaluated using enCore software, version 17 (GE HealthCare).^21^

### Patient-Reported Outcome and Experience Measures

Gastrointestinal tract function was evaluated by the Gastrointestinal Symptom Rating Scale (GSRS)^22^ and a bowel-function questionnaire.^23^ Bothersome symptoms were defined as a GSRS score of 3 or more.^24^ Health-related quality of life was evaluated by the generic 36-Item Short Form Health Survey (SF-36), version 2.0, with 4-week recall,^25,26,27^ and obesity-specific quality of life was evaluated by the Obesity-Related Problems (OP) Scale.^28^ The OP Scale scores were categorized as mild, moderate, or severe impairment based on the mean score (in percentages). The Three-Factor Eating Questionnaire^29^ was used to evaluate eating behavior. A nonvalidated 3-question patient-reported experience measure was used with a visual scale of 0 to 10 points, higher scores indicating a higher degree of agreement to questions asked (eTable 8 in Supplement 1).

### Sample Size and Power Estimation

A total of 26 study participants in each group resulted in 80% power to detect a significant difference (2-sided *P* ≤ .05) between the groups in terms of weight loss and BMI reduction 2 years after surgery.^14,30^ In total, 60 participants underwent study procedures.

### Statistical Analysis

Linear mixed models were fitted for continuous variables measured more than 2 times (weight, BMI, blood pressure, and blood or serum analyte levels). Fixed effects were treatment, time, and treatment × time interaction. Time was modeled as a piecewise linear function with knots at 6 months and 1, 2, and 5 years after surgery. The models included a random intercept. Based on the fitted models, we estimated mean values with 95% CIs for each treatment group at baseline and 1, 2, 5, and 10 years after surgery. We estimated the change from baseline to 10 years in each group and the between-group difference in changes from baseline to 10 years with 95% CIs. Continuous variables were also analyzed using independent sample *t* tests for data with an approximately normal distribution and Mann-Whitney tests for data that deviated from the normal distribution. Categorical variables (comorbidities and adverse effects) were compared using Pearson χ^2^ tests and Fisher mid-*P* tests for expected counts less than 5.Analyses were performed using SPSS, version 26 (IBM Corp), and Stata for Windows, version 17 (StataCorp LLC). Statistical significance for all tests was set at *P* ≤ .05. All reported *P* values are 2-tailed.

## Results

**Figure 1.**
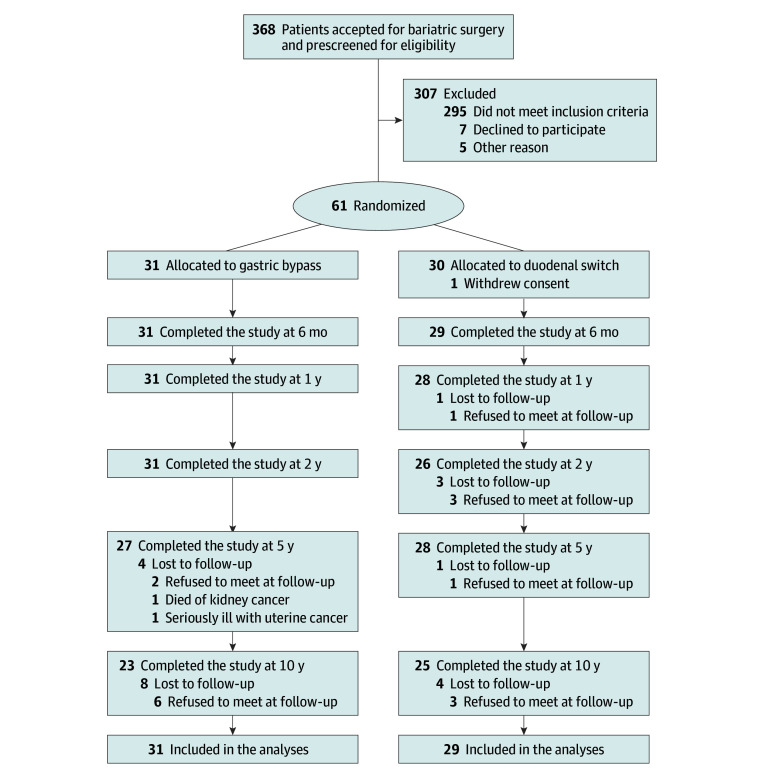
Flowchart of Patients Through Recruitment and Follow-Up

In total, 48 of 60 patients (80%) attended consultations 10 years or more after surgery (23 in the RYGB group and 25 in the DS group) (Figure 1). The mean (SD) age was 48.0 (6.0) years overall, 47.8 (6.9) years for RYGB, and 48.5 (5.3) for DS. A total of 35 patients (73%) were women and 13 (27%) were men, with 18 women (78%) and 5 men (22%) in the RYGB group and 17 women (68%) and 8 men (32%) in the DS group. Median follow-up was 12 (range, 9-13) years. Patient characteristics are presented in Table 1 and eTable 2 in Supplement 2.

**Table 1.** Cardiometabolic Risk Factors in 60 Patients Randomized to RYGB or DS From Baseline to 10 Years After Surgery Using Linear Mixed Models

Risk factor by procedure	Measurement time, mean (95% CI)			Change from baseline to 10 y	Between-group difference in changes from baseline to 10 y	*P* value
	Baseline^a^	5 y^b^	10 y^c^			
Weight, kg						
RYGB	161.1 (154.6 to 167.6)	119.5 (111.9 to 127.2)	128.0 (118.7 to 137.4)	−33.1 (−41.0 to −25.1)	27.5 (16.3 to 38.8)	<.001
DS	162.7 (156.0 to 169.5)	96.6 (88.8 to 104.4)	102.1 (92.6 to 111.6)	−60.6 (−68.6 to −52.6)		
BMI						
RYGB	54.5 (53.3 to 55.6)	40.6 (38.7 to 42.5)	43.5 (40.7 to 46.2)	−11.0 (−13.7 to −8.3)	9.3 (5.4 to 13.1)	<.001
DS	55.3 (54.1 to 56.5)	33.4 (31.5 to 35.2)	35.0 (32.3 to 37.8)	−20.3 (−23.0 to −17.6)		
Waist circumference, cm						
RYGB	150 (146 to 154)	121 (116 to 126)	121 (115 to 128)	−29 (−35 to −23)	17 (9 to 25)	<.001
DS	153 (149 to 157)	107 (102 to 112)	107 (101 to 113)	−46 (−52 to −40)		
Systolic BP, mm Hg						
RYGB	134 (128 to 140)	126 (119 to 132)	136 (130 to 141)	2 (−4 to 8)	9 (1 to 18)	.02
DS	138 (132 to 144)	123 (117 to 129)	131 (125 to 136)	−8 (−13 to −2)		
Diastolic BP, mm Hg						
RYGB	83 (79 to 86)	78 (73 to 82)	81 (77 to 84)	−2 (−6 to 2)	6 (0 to 12)	.052
DS	88 (84 to 92)	78 (73 to 82)	80 (77 to 84)	−8 (−12 to −4)		
Glucose level, mg/dL^d^						
RYGB	106 (101 to 114)	106 (99 to 115)	106 (97 to 115)	0 (−11 to 9)	18 (5 to 31)	.007
DS	110 (103 to 117)	85 (77 to 94)	92 (83 to 101)	−20 (−29 to −9)		
HbA_1c_ level, %^e^						
RYGB	6.1 (5.7 to 6.5)	5.5 (5.0 to 5.9)	5.7 (5.4 to 6.0)	−0.4 (−0.8 to 0.0)	0.9 (0.3 to 1.5)	.005
DS	6.3 (5.8 to 6.7)	5.0 (4.6 to 5.4)	5.0 (4.7 to 5.3)	−1.3 (−1.7 to −0.8)		
HbA_1c_ level, mmol/mol^f^						
RYGB	43 (38 to 48)	36 (31 to 41)	39 (35 to 42)	−4 (−9 to 0.5)	10 (3 to 17)	.006
DS	45 (40 to 50)	31 (26 to 35)	31 (28 to 34)	−14 (−19 to −9)		
Total cholesterol level, mg/dL^g^						
RYGB	178 (170 to 185)	181 (170 to 193)	174 (166 to 185)	−4 (−12 to 8)	46 (31 to 58)	<.001
DS	181 (174 to 193)	135 (123 to 147)	135 (123 to 143)	−46 (−58 to −39)		
HDL cholesterol level, mg/dL^h^						
RYGB	43 (43 to 46)	66 (58 to 70)	62 (54 to 66)	19 (15 to 23)	8 (4 to 15)	.03
DS	39 (35 to 43)	54 (46 to 58)	50 (46 to 58)	12 (8 to 15)		
LDL cholesterol level, mg/dL^i^						
RYGB	104 (97 to 112)	100 (93 to 108)	93 (85 to 100)	−12 (−19 to −4)	31 (19 to 43)	<.001
DS	104 (100 to 116)	70 (62 to 77)	66 (58 to 73)	−39 (−46 to −31)		
Triglyceride level, mg/dL^j^						
RYGB	150 (133 to 168)	89 (71 to 106)	106 (89 to 115)	−23 (−31 to −12)	44 (18 to 71)	.001
DS	177 (159 to 195)	71 (53 to 89)	80 (71 to 97)	−97 (−115 to −80)		

Abbreviations: BMI, body mass index (calculated as the weight in kilograms divided by the height in meters squared); BP, blood pressure; DS, duodenal switch; HbA_1c_, hemoglobin A_1c_; HDL, high-density lipoprotein; RYGB, Roux-en-Y gastric bypass.

SI conversion factors: To convert cholesterol to mmol/L, multiply by 0.0259; glucose to mmol/L, multiply by 0.0555; triglycerides to mmol/L, multiply by 0.0113.

^a^ Includes 31 patients in the RYGB group and 29 in the DS group.

^b^ Includes 27 patients in the RYGB group and 28 in the DS group.

^c^ Includes 23 patients in the RYGB group and 25 in the DS group.

^d^ Reference range was 72 to 108 mg/dL.

^e^ Reference range was 4.0% to 6.0%. Values were converted to millimoles per mole (when missing), then analyzed with linear mixed models.

^f^ Reference range was 20 to 42 mmol/mol.

^g^ Reference range was 127 to 266 mg/dL.

^h^ Reference range was 31 to 81 mg/dL.

^i^ Reference range was 73 to 185 mg/dL.

^j^ Reference range was 44 to 230 mg/dL.

### Body Weight and BMI

**Figure 2.**
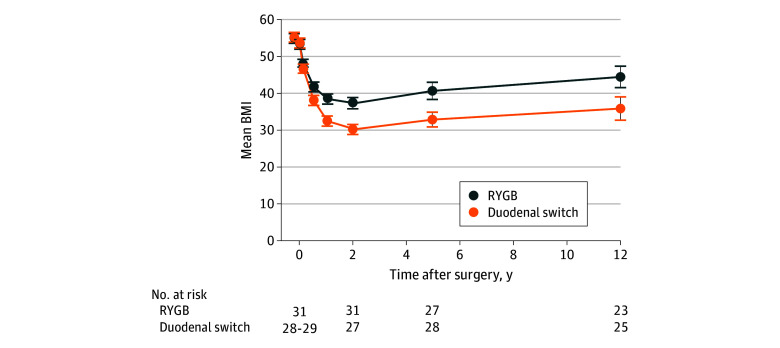
Body Mass Index (BMI) for 60 Patients Undergoing Roux-en-Y Gastric Bypass (RYGB) and Duodenal Switch From Baseline to 10 Years After Surgery The number of patients in the DS group varied in the first year, with 29 at baseline, 28 at 6 weeks, 29 at 6 months, and 28 at 1 year. The number of patients in the RYGB group was 31 at baseline, 6 weeks, 6 months, and 1 year. Error bars indicate 95% CIs.

At follow-up, the mean BMI reductions were 11.0 (95% CI, 8.3-13.7) for the RYGB group and 20.3 (95% CI, 17.6-23.0) for the DS group, with a mean between-group difference of 9.3 (95% CI, 5.4-13.1; *P* < .001). Total weight loss was 20.0% (95% CI, 15.3%-24.7%) for the RYGB group and 33.9% (95% CI, 27.8%-40.0%) for the DS group (*P* = .001). Weight and BMI development over 10 years are presented in Table 1. Both procedures induced maximal weight loss at approximately 2 years with subsequent weight regain (Figure 2). Mean weight regain from the nadir was 19.8 (95% CI, 12.0-27.6) kg for the RYGB group and 15.9 (95% CI, 8.6-23.2) kg for the DS group, with a mean between-group difference of 3.9 (95% CI, 6.5-14.3) kg (*P* = .45). Mean BMI at follow-up was 44.5 (95% CI, 41.6-47.4) for the RYGB group and 35.9 (95% CI, 32.8-39.1) for the DS group, with a mean between-group difference of 8.6 (95% CI, 4.4-12.8) (*P* < .001). The proportions of patients with BMI of 40 or greater were 16 of 23 (70%) after RYGB and 8 of 25 (32%) after DS (*P* = .009).

### Comorbidity and Cardiometabolic Measures

There were no significant differences in the prevalence of type 2 diabetes, dyslipidemia, or metabolic syndrome between the groups at follow-up (eTable 2 in Supplement 2). Mean serum lipid levels improved more in the DS group except for high-density lipoprotein cholesterol levels, which increased more after RYGB (Table 1). Mean HbA_1c_ level was 5.7% (95% CI, 5.4%-6.0%) for the RYGB group and 5.0% (95% CI, 4.7%-5.3%) for the DS group (*P* = .005) 10 years after surgery, corresponding to 39 (95% CI, 35-42) mmol/mol for RYGB and 31 (95% CI, 28-34) mmol/mol for DS (*P* = .006) (Table 1).

### Adverse Events, Hospital Admissions, and Subsequent Surgery

The total number of adverse events was 97 after RYGB and 135 after DS (*P* = .02), with a mean (SD) number of adverse events per patient of 3.1 (2.5) and 4.6 (2.6), respectively (*P* = .01) (eTable 3 in Supplement 1). After RYGB, 21 of 31 patients (68%) were admitted to the hospital for any reason, and 18 of 29 (62%) after DS (*P* = .65). The number of hospital admissions and abdominal surgical procedures were similar. Seven of 31 patients (23%) in the RYGB group and 11 of 29 (38%) in the DS group underwent body reconstructive surgery (*P* = .29).

More patients underwent cholecystectomies after DS (11 in the DS group vs 4 in the RYGB group; *P* = .03). Four of 29 patients (14%) developed protein-caloric malnutrition after DS compared with none after RYGB (*P* = .02). Three of these 4 patients (10% of the total) underwent surgery with elongation of the common channel (3.5, 4, and 5 years after DS). Symptoms of gastroesophageal reflux were more prevalent after DS (10 vs 4 patients; *P* = .05). One patient died 13 years after DS, and 2 patients died 4 and 5 years after RYGB.

### Nutritional Status

The mean levels of vitamins A and E were lower after DS throughout the study period (Table 2). On average, there was no difference in 25-hydroxyvitamin D (25[OH]D) levels between groups, but 25(OH)D deficiency was observed in 9 of 23 patients (39%) after RYGB and in 19 of 25 (76%) after DS (*P* = .008) (eTable 2 in Supplement 2). Vitamin B_1_ (thiamine) levels were lower after DS in mixed model analysis. Mean levels of hemoglobin, albumin, and ionized calcium were similar between groups (Table 2). Secondary hyperparathyroidism was more prevalent after DS than RYGB (15 vs 7; *P* = .02). Among patients in the DS group, 21 of 25 (84%) had 1 or more vitamin deficiency compared with 11 of 23 (48%) after RYGB (*P* = .005) (eTable 2 in Supplement 2). Use of supplements is presented in eTable 4 in Supplement 2. Nutritional variables are presented in Table 2.

**Table 2.** Nutritional Variables in 60 Patients Randomized to RYGB or DS From Baseline to 10 Years After Surgery Using Linear Mixed Models

Nutritional variable by procedure	Measurement time, mean (95% CI)			Change from baseline to 10 y	Between-group difference in changes from baseline to 10 y	*P* value
	Baseline	5 y	10 y			
Vitamin A level, μg/dL^a^						
RYGB	49 (43 to 52)	49 (46 to 54)	49 (43 to 52)	0 (−3 to 6)	11 (6 to 20)	<.001
DS	46 (43 to 52)	32 (29 to 37)	34 (29 to 40)	−11 (−17 to −6)		
Vitamin B_1_ level, μg/dL^b^						
RYGB	4.1 (3.8 to 4.4)	4.6 (4.3 to 5.0)	5.2 (4.7 to 5.6)	1.0 (0.5 to 1.5)	−0.7 (−1.4 to −0.1)	.03
DS	4.1 (3.8 to 4.4)	5.3 (5.0 to 5.7)	5.8 (5.4 to 6.4)	1.8 (1.3 to 2.3)		
Vitamin B_6_ level, ng/mL^c^						
RYGB	6.8 (4.4 to 9.1)	8.8 (5.7 to 12.0)	8.7 (5.1 to 12.2)	1.9 (−1.9 to 5.7)	−1.04 (−6.50 to 4.40)	.71
DS	6.8 (4.4 to 9.3)	12.2 (9.1 to 15.4)	9.8 (6.2 to 13.4)	3.0 (−0.9 to 6.9)		
Vitamin B_9_ level, ng/mL^d^						
RYGB	6.6 (5.1 to 8.2)	7.5 (5.9 to 9.0)	8.8 (7.2 to 10.5)	2.2 (−0.1 to 4.4)	−2.2 (−5.1 to 1.4)	.26
DS	6.0 (4.4 to 7.6)	9.8 (8.3 to 11.3)	10.0 (8.4 to 11.7)	4.0 (1.7 to 6.3)		
Vitamin B_12_ level, pg/mL^e^						
RYGB	398 (263 to 533)	648 (508 to 786)	721 (539 to 903)	323 (107 to 538)	92 (−211 to 397)	.55
DS	541 (403 to 678)	632 (495 to 770)	771 (591 to 950)	230 (16 to 446)		
25-Hydroxyvitamin D level, ng/mL^f^						
RYGB	19.9 (17.2 to 22.6)	17.9 (14.3 to 21.5)	22.3 (18.6 to 26.0)	2.4 (−1.0 to 5.9)	4.0 (−0.9 to 8.9)	.11
DS	20.5 (17.7 to 23.3)	11.8 (8.1 to 15.5)	19.0 (15.2 to 22.7)	−1.6 (−5.0 to 1.9)		
Vitamin E level, μg/mL^g^						
RYGB	1.7 (1.6 to 1.8)	2.0 (1.9 to 2.1)	2.3 (2.1 to 2.5)	0.6 (0.4 to 0.8)	0.6 (0.3 to 0.9)	<.001
DS	1.7 (1.6 to 1.8)	1.9 (1.7 to 2.0)	1.7 (1.5 to 1.8)	0.0 (−0.2 to 0.2)		
Parathyroid hormone level, pg/mL^h^						
RYGB	5.8 (4.5 to 7.0)	8.4 (6.0 to 10.9)	7.7 (3.8 to 11.6)	1.9 (−1.9 to 5.8)	−5.8 (−11.3 to −0.4)	.04
DS	6.2 (4.9 to 7.6)	15.5 (13.0 to 18.0)	14.0 (10.1 to 17.9)	7.8 (3.9 to 11.7)		
Albumin level, g/dL^i^						
RYGB	4.3 (4.2 to 4.5)	4.4 (4.2 to 4.6)	4.2 (4.1 to 4.3)	−0.1 (−0.3 to 0.1)	0.0 (−0.2 to 0.3)	.79
DS	4.3 (4.1 to 4.4)	4.3 (4.1 to 4.5)	4.1 (4.0 to 4.2)	−0.1 (−0.3 to 0.0)		
Hemoglobin level, g/dL^j^						
RYGB	1.4 (1.4 to 1.5)	1.4 (1.3 to 1.4)	1.3 (1.3 to 1.4)	−0.1 (−0.2 to 0.0)	0.0 (−0.1 to 0.1)	.65
DS	1.4 (1.4 to 1.5)	1.3 (1.3 to 1.34)	1.3 (1.3 to 1.4)	−0.1 (−0.2 to −0.1)		
Ionized calcium level, mg/dL^k^						
RYGB	5.00 (4.92 to 5.08)	4.76 (4.68 to 4.80)	4.76 (4.72 to 4.84)	−0.24 (−0.32 to −0.16)	0.08 (0.00 to 0.20)	.08
DS	5.00 (4.92 to 5.04)	4.60 (4.56 to 4.68)	4.68 (4.60 to 4.72)	−0.32 (−0.40 to −0.24)		

Abbreviations: DS, duodenal switch; LDL, low-density lipoprotein; RYGB, Roux-en-Y gastric bypass.

SI conversion factors: To convert albumin to g/L, multiply by 10; hemoglobin to g/L, multiply by 10.0; ionized calcium to mmol/L, multiply by 0.25; parathyroid hormone to pmol/L, multiply by 9.43; vitamin A to μmol/L, multiply by 0.0349; vitamin B_1_ to nmol/L, multiply by 29.6; vitamin B_6_ to nmol/L, multiply by 4.046; vitamin B_9_ to nmol/L, multiply by 2.266; vitamin B_12_ to pmol/L, multiply by 0.7378; vitamin E to μmol/L, multiply by 2.322; 25-hydroxyvitamin D to nmol/L, multiply by 2.496.

^a^ Reference range was 20 to 80 μg/dL.

^b^ Reference range was 3.2 to 6.8 μg/dL.

^c^ Reference range was 3.7 to 39.6 ng/mL.

^d^ Reference level was less 3 ng/mL.

^e^ Reference range was 271 to 1355 pg/mL.

^f^ Reference level was 14.82 to 52.48 ng/mL.

^g^ Reference range was 8.18 to 21.53 μg/mL.

^h^ Reference range was 11.3 to 67.0 pg/mL.

^i^ Reference range was 3.8 to 5.2 g/dL.

^j^ Reference range was 1.34 to 1.70 g/dL.

^k^ Reference range was 4.60 to 5.32 mg/dL.

### Bone Mineral Density and Serum Bone Markers

From 5 to 10 years, aBMD decreased 7.1% in total, 8.0% in the spine, and 6.6% in the femoral neck after DS; after RYGB, aBMD decreased 0.8% in total, 1.7% in the spine, and 2.0% in the femoral neck. Mean total aBMD and mean femur total aBMD were lower for DS at 5 and 10 years. At 10 years, aBMD in L1 to L4 and aBMD in the femoral neck were lower in the DS group (eTable 5 and eFigure 1 in Supplement 2). The prevalence of osteoporosis was 4 in the RYGB group vs 10 in the DS group (*P* = .15); prevalence of osteopenia was 7 in the RYGB group vs 11 in the DS group (*P* = .54) (eTable 3 in Supplement 2). Analyzing osteoporosis and osteopenia combined, there was a higher incidence of reduced bone mass in the DS group (11 in the RYGB group vs 21 in the DS group *P* = .004).

At 5 years, the mean CTX-1 level was within reference range after RYGB and higher after DS (mean between-group difference, 0.25 [95% CI, 0.48-0.02] μg/L; *P* = .04). Mean P1NP levels were elevated for both groups, but higher after DS (mean between-group difference, 47.7 [95% CI, 70.3-25.0] μg/L; *P* < .001). There were no between-group differences for mean CTX-1 and P1NP levels at 10 years (eTable 6 in Supplement 2).

### Gastrointestinal Tract Function

Scores on the GSRS for abdominal pain, indigestion, diarrhea, and constipation were comparable except for higher reflux score for DS at 10 years. More patients had bothersome symptoms of reflux and diarrhea after DS (eTable 7 in Supplement 2).

Frequencies of defecations and anal leakage of flatus and feces were similar across groups. Patients in the RYGB group reported more days over the last month without defecation (9 in the RYGB group vs 2 in the DS group; *P* = .005) during the last month. More patients in the DS group reported loose stools (4 in the RYGB group vs 12 in the DS group; *P* = .02).

### Quality of Life

Scores on the SF-36 were similar across groups at 10 years. The SF-36 domain scores during follow-up are shown in eFigure 2 in Supplement 2.

**Figure 3.**
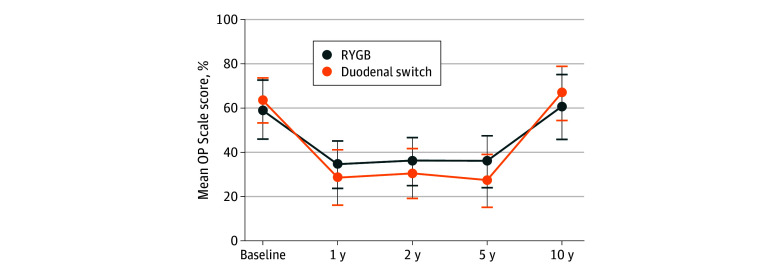
Obesity-Related Problem (OP) Scale for 60 Patients Scores are given at baseline and 1, 2, 5, and 10 years after surgery for Roux-en-Y gastric bypass (RYGB [n = 31]) and duodenal switch (n = 29). One hundred indicates worst possible psychosocial impairment; 0, no psychosocial impairment (<40% indicates mild psychosocial impairment; 40%-59%, moderate psychosocial impairment; and ≥60%, severe psychosocial impairment). Error bars indicate 95% CIs.

Severe obesity–related psychosocial impairment (OP Scale scores [Figure 3]) was reported in 15 patients in the RYGB group and 16 in the DS group at baseline (*P* = .60) and in 14 in the RYGB group and 14 in the DS group at 10 years (*P* = .83). Prevalence of uncontrolled and emotional eating were similar between groups at 10 years, but patients in the RYGB group displayed a higher degree of cognitive constraint eating, with 46% (95% CI, 37%-54%) for the RYGB group and 33% (95% CI, 24%-41%) for the DS group (*P* = .04). There were no statistically significant differences regarding patient-reported experience measurements of adverse effects and complications, satisfaction with having had the surgery, or whether they would recommend the surgery to others (eTable 8 in Supplement 2).

## Discussion

This comprehensive long-term evaluation shows that DS induced significantly higher weight loss compared with RYGB in patients with an initial BMI of 50 to 60. At 10 years, 70% of patients with RYGB still had a BMI of 40 or greater. The DS procedure conferred some benefits related to lipid and glycemic profiles, but was associated with more adverse effects, more vitamin deficiencies, and more malabsorption. Quality-of-life scores were similar, with trajectories approaching baseline values for several measures at final follow-up.

Several studies report more weight loss after DS, but typically without a randomized design or with more heterogeneous populations not exclusively with BMI of 50 or greater.^6,7,8,9,11,12,13^ In a retrospective study of 77 406 patients undergoing RYGB and 1540 undergoing DS with initial mean BMI of 48 and 52, respectively, a greater weight loss and more improvements in comorbidities were observed after DS.^5^ In a prospective series (initial BMI, ≥50), superior weight loss was reported after DS at 36 months.^10^ One randomized clinical trial including 34 patients reported weight loss to 17 years after DS comparable with our findings.^3,31^ This study, however, was prematurely closed as patients declined randomization. Long-term outcomes, including weight loss and comorbidities, were based on data reported by the patients.

Both RYGB and DS induced similar improvements in obesity-related comorbidities in terms of number of patients with type 2 diabetes, dyslipidemia, and metabolic syndrome. Blood and serum lipid and HbA_1c_ levels, however, improved more after DS, as did mean waist circumference. Whether such differences, mainly within the reference range, may translate into long-term cardiovascular health benefits is unclear.

To our knowledge, this is the first study to compare bone health after RYGB and DS within a randomized clinical setting. Patients undergoing DS had lower bone density 10 years after surgery. There was a clinically significant reduction in aBMD at all sites from 5 to 10 years after DS but not after RYGB. Elevated bone turnover markers in both groups may indicate increased bone resorption. Combined, these results warrant concern for bone health, especially after DS. When comparing patients with osteoporosis and osteopenia, combined bone mass was significantly lower in the DS group. Increased prevalence of vitamin D deficiency and secondary hyperparathyroidism after DS may be contributing factors.

The number of adverse effects were higher after DS than RYGB, but there was no difference in the number of hospital admissions. A higher rate of cholecystectomies after DS is also reported by others.^3^ At 10 years, we found lower levels of fat-soluble vitamins A and E after DS. Mean vitamin D levels were similar between groups. However, there was a higher prevalence of vitamin D deficiency after DS (9 patients in the RYGB group vs 19 in the DS group; *P* = .005). Protein caloric deficiency in 4 patients (14%), including 3 receiving revisional surgery due to malnutrition, developed exclusively after DS. These observations corroborate findings reported by others.^3,32^ Vitamin levels have not been a concern when studied in a general population undergoing RYGB in one of the participating institutions.^33^

More patients reported bothersome reflux and diarrhea symptoms after DS. Results related to gastroesophageal reflux are in line with previous findings demonstrating more reflux after sleeve gastrectomy than RYGB.^34^

Analyses of obesity-related psychosocial problems displayed similar trends, with improvements from baseline to 5 years after surgery, and subsequent increase in scores almost to baseline values at 10 years or later (Figure 3). Generic quality-of-life analyses (SF-36) showed similar trends, with initial improvements attenuating over time in most of the 8 domains. Both groups reported similar levels of satisfaction with having undergone bariatric surgery at 10 years.

### Strengths and Limitations

Strengths of the study include the randomized design, the long follow-up with an 80% physical attendance rate, and the comprehensive evaluation at 10 years. Limitations include the limited sample size, which warrants caution in assessment of several of the outcome measures. A postoperative supplement regimen better adapted to the higher risk of malabsorption after DS, including higher doses of vitamin supplements, could have compensated for malabsorption in particular of fat-soluble vitamins after DS.

## Conclusions

Although DS enables a greater BMI reduction over time and some cardiometabolic benefits, this comes at a cost regarding nutritional deficiencies and adverse effects. The balance between potential benefits and potential adverse effects is delicate, and a larger weight loss per se may not justify an increased risk. Despite the initial hypothesis in this randomized clinical trial that DS was a superior option to RYGB in patients with a BMI of greater than 50, we have ultimately concluded with the opposite, and DS with a short common channel (<200 cm) is currently no longer used in our institutions. For strictly selected patients with particularly high BMI and related comorbidity, the risk and benefit balance may justify the use of DS in experienced centers. The developing landscape of obesity treatment may suggest prioritizing bariatric techniques with the most optimal risk-benefit profile and adding modern weight loss medications when needed.

## Notes

Supplement 1.
Trial Protocol

Supplement 2. **eTable 1.** Trial Definitions of Comorbidities and Their Resolutions **eTable 2.** Comorbidities and Vitamin Deficiencies 10 Years or More After Roux-en-Y Gastric Bypass (RYGB) and Duodenal Switch (DS) Surgery **eTable 3.** Adverse Events and Effects 30 Days After Surgery to 10 Years After Roux-en-Y Gastric Bypass (RYGB) and Duodenal Switch (DS) **eTable 4.** Vitamin and Mineral Supplementation Reported at Baseline and 5 and 10 Years After Roux-en-Y Gastric Bypass (RYGB) and Duodenal Switch (DS) Surgery **eTable 5.** Areal Bone Mineral Density (aBMD) for Patients 5 and 10 Years After Roux-en-Y Gastric Bypass (RYGB) and Duodenal Switch (DS) **eTable 6.** Serum Bone Turnover Markers 5 and 10 Years After Roux-en-Y Gastric Bypass (RYGB) and Duodenal Switch (DS) **eTable 7.** Gastrointestinal Symptoms Rating Scale (GSRS) **eTable 8.** Patient-Reported Experience Measures (PREM) 10 Years or More for 44 Patients After Roux-en-Y Gastric Bypass (RYGB [n = 21]) and Duodenal Switch (DS [n = 23]) Surgery **eFigure 1.** Areal Bone Mineral Density (aBMD) Scores From Dual Energy X-Ray Absorptiometry (DEXA) Scans for 60 Patients 5 and 10 Years After Roux-en-Y Gastric Bypass (n = 31) and Duodenal Switch (n = 29) Surgery **eFigure 2.** Short Form (SF-36) Questionnaire for 60 Patients at Baseline, 5 and 10 Years After Roux-en-Y Gastric Bypass (RYGB) (n = 31) and Duodenal Switch (DS) (n = 29)

Supplement 3.
Data Sharing Statement
