# Supplementary material for: Ten-Year Outcomes Following Roux-en-Y Gastric Bypass vs Duodenal Switch for High Body Mass Index: A Randomized Clinical Trial
Source: JAMA Netw Open. 2024 Jun 3;7(6):e2414340. doi: 10.1001/jamanetworkopen.2024.14340 (PMC11148687; doi:10.1001/jamanetworkopen.2024.14340)
Supplement: Supplement 1. — Trial Protocol [file jamanetwopen-e2414340-s001.pdf]

Oslo, 24 Feb 2006

Research protocol

Laparoscopic gastric bypass or laparoscopic biliopancreatic diversion with duodenal switch in the treatment of extreme obesity (BMI 50 – 60 kg/m²): A prospective, randomized two-center trial

Participating hospitals:

Oslo University Hospital Aker, Oslo, Norway

Sahlgrenska University Hospital, Gothenburg, Sweden

Principal investigators:

Torsten Olbers, MD, PhD

Jon Kristinsson, MD, PhD

Collaborators at Oslo University Hospital Aker:

Torgeir T. Søvik, MD, PhD candidate

Erlend T. Aasheim, MD, PhD candidate

Tom Mala, MD, PhD

Professor Arild Nesbakken, MD, PhD

Carl Fredrik Schou, MD

Sigrun Henjum, RD

Ann Steen, RN, research coordinator

Collaborators at Sahlgrenska University Hospital:

Osama Taha, MD, PhD candidate

Sofia Björkman, RD

My Engström, RN, research coordinator

Professor Hans Lönroth, MD, PhD

1. Background

Body mass index (BMI, kg/m^2) is the most commonly used measure for classifying overweight and obesity. *Morbid obesity* may be defined as having a BMI above 40, or a BMI of over 35 with obesity related comorbidity (i.e. conditions that may be ameliorated or resolved after surgically induced weight loss).[1] The prevalence of obesity is increasing in Norway. Among adults, an estimated 13.500 persons currently have a BMI of over 40.

Morbid obesity is associated with psychosocial problems, reduced work capacity, and increased morbidity and mortality from conditions such as cardiovascular disease, diabetes, and asthma. At present, bariatric surgery is the only treatment option inducing long-term weight reduction in morbidly obese patients.[2;3]

Of bariatric procedures currently performed in Norway, biliopancreatic diversion with duodenal switch (duodenal switch) is perceived to be associated with the greatest degree of weight reduction, and possibly, the best quality of eating after surgery.[3] However, both short and long-term morbidity appears to be somewhat higher than after laparoscopic Roux-en-Y gastric bypass (gastric bypass), which is the most commonly performed laparoscopic bariatric operation for morbid obesity in patients with a BMI in the range of 35 to 50.

In the gastric bypass procedure, the small bowel is arranged in such a fashion that food is diverted past the upper part of the small bowel.[4] The length of this small bowel bypass is usually in the range of 50 to 150 cm. However, the ideal bypass length has not been determined.

Intestinal uptake of nutrients occurs chiefly after the junction of the small bowel carrying food (the alimentary limb) and the biliopancreatic limb, which transports digestive juices. This distal part of the small bowel is called the “common channel”, and its length is inversely associated with the extent of the bypass. In extremely obese patients (BMI over 50), gastric bypass performed with a short small bowel bypass provides insufficient weight reduction for a high proportion of patients. However, an extended small bowel bypass, and thus a reduced length of the common channel, is associated with increased weight reduction for this group of patients, probably with an acceptable risk of malnutrition.[5;6]

The duodenal switch procedure involves the resection of ¾ of the stomach, a small bowel bypass, and a very short common channel.[7] This procedure has been performed as open surgery for more than 20 years, but is now feasible by laparoscopy.[8] The procedure confers good weight reduction, and some health care providers prefer this operation in super-obese patients. Duodenal switch performed by

laparoscopy is technically more challenging than laparoscopic gastric bypass, and is possibly associated with a higher morbidity rate, in particular due to long-term complications such as exaggerated malabsorption.

It is not known whether gastric bypass or duodenal switch is the most suitable bariatric surgical technique in super-obesity, and no randomized trials have been performed to address this. Thus, we consider this research project relevant and important.

Feasibility of the study:

Both gastric bypass and duodenal switch are performed at Oslo University Hospital Aker (OUH) and at Sahlgrenska University Hospital (SU) with standardized surgical techniques.

The principal investigator at SU holds a PhD in surgical treatment of morbid obesity. The registered dietician at SU involved in the study has co-authored several papers published in peer-reviewed journals on similar topics. The collaborators at OUH have not previously conducted research in the field of bariatric surgery, but have substantial general research experience. Collaborators at OUH are also currently accumulating clinical experience in this field and have participated on several international scientific congresses.

In Norway, OUH has a regional responsibility for the treatment of morbid obesity. Thus, we consider it possible to recruit a sufficient number of study participants over a short period of time. The department has the resources to carry out the practical parts of the study by personnel already employed and the financial resources for the research are available at the hospital.

2. Aims of the study

To compare treatment outcome in super-obese (BMI 50-60), aged 20-50 years at inclusion, after:

- Laparoscopic gastric bypass with a long (150 cm) alimentary limb (long limb laparoscopic gastric bypass).
- Laparoscopic biliopancreatic diversion with duodenal switch (alimentary limb 200 cm and common channel 100 cm).

Primary end point: Long-term results regarding weight reduction and weight stability

Secondary end points: Long-term results regarding quality of eating and quality of life
Per- and postoperative complications
Postoperative mobilization and length of hospital stay
Long-term complications (including surgical revisions)
Changes in body composition
Nutritional deficiencies after surgery
Demand for hospital services (i.e. operating costs, outpatient visits)
Long-term results regarding eating disorders
Changes in comorbidities

Based on the same data set, more detailed studies regarding the nutritional consequences of surgery for obesity will be carried out. This is defined in more detail in a separate research protocol with the principal research fellow Erlend T. Aasheim and professor Thomas Bøhmer, Department of Medicine, Oslo University Hospital Aker.

Hypotheses:

Both surgical techniques induce similar (not significantly different) weight loss and late weight regain.
The quality of life is similar in the two groups.
The quality of eating is similar in the two groups.
The incidence of perioperative and late complications are similar.
The methods are associated with similar rates of nutritional deficiencies and gastrointestinal problems.
Body composition (loss of fat and muscle mass) is changed equally in the two groups.

Alternative hypotheses:

Duodenal switch leads to a 10-20% greater weight loss (after two years) than does gastric bypass.
Weight regain from two years after surgery is greater after gastric bypass than after duodenal switch.
Duodenal switch patients experience more gastrointestinal side effects than does patients after gastric bypass.
Duodenal switch patients experience more nutritional disorders.

3. Material

Inclusion criteria:

Patients scheduled for surgery for morbid obesity with a BMI of 50 – 60 at referral (48 – 62 at inclusion).
Age 20 to 50 years.
Signed informed consent.

Exclusion criteria:

Previous bariatric surgery.
Previous major abdominal surgery.
Other serious illnesses (such as malignancy or severe cardiopulmonary diseases).
Chronic treatment with oral steroids.
Patients with a lack of ability to cooperate in or complete the study.

4. Methods

Study design:

Two-center, prospective, randomized trial.
Relevant parameters are registered prospectively in designated form (Case Record Form).
Each patient is his own control regarding the above-mentioned end points.

Analytical methods:

Weight loss evaluated before and 1½, 6, 12, 24 and 60 months after surgery:
Weight, BMI, and percentage weight lost.

Body composition evaluated before and 12 and 24 months after surgery:
Waist and hip circumferences and sagittal abdominal diameter.
Segmental bioelectrical impedance analysis in Norwegian participants. 40K spectroscopy in Swedish participants.

Quality of life evaluated by the following questionnaires before and 12, 24 and 60 months after surgery:
Short Form 36 health survey (SF-36).
The Obesity-related problems scale (OP-scale).
Gastrointestinal quality of life (GSRS).
Questionnaire on bowel function ("Frågeformulär tarmfunktion").

Eating pattern (measured at similar time points):
Three factor eating questionnaire (TFEQ).
Four-day dietary record.
Additional questions about diet and supplements.

Health economics:
The use of disposable surgical equipment.
Lengths of surgery and anesthesia.
Lengths of stay in post-operative department and in intensive care unit.
Length of hospital stay (expenses are converted into costs in NOK or Euros by current method applied at Sahlgrenska).
Number of visits in primary care and outpatient clinics because of obesity - before and after surgery.

Associated diseases evaluated before and 12, 24 and 60 months after surgery:

Diabetes:	Measurement of fasting plasma glucose and insulin and record of any use of antidiabetic drugs.
Hypertension:	Measurement of blood pressure and the need for medication.
Dyslipidemia:	Measurement of lipids and the need for medication.

Complications:
Recording of early complications (within 30 days after surgery) such as anastomotic leak, hemorrhage demanding surgical intervention, pulmonary embolism, abscess, reoperations, and death etc. Further registration of late (>30-day) complications at outpatient visits.

Data

Specially designated case record forms (CRFs) will be used for continuous registration.

Data from the case record forms will be entered into the database and then be coded (pseudonymized).

One person at each hospital is responsible for the recording of information into the database, and only this person is familiar with the code key. Approval from The Data Inspectorate for the registration of information for all patients operated for obesity at OUH is granted.

Statistical methods

For between-group comparisons, Mann-Whitney U test and Wilcoxon's test will be used for non-parametric data, and Student's t test and paired *t* test for parametric analysis. Dichotomous outcome variables will be analyzed with chi-square test or Fisher's exact test as appropriate.

Sample size

Sample size calculation is based on change in weight. In a retrospective study from Sahlgrenska, mean (SD) reductions in BMI for super-obese patients 3 years after surgery were 24.9 (5.0) after duodenal switch (n=13) and 18.0 (6.7) after gastric bypass (n=19). Thus, 60 study participants would give >90% power to detect a difference in the change in BMI between groups (2-sided *t* test, *P*=.05).

Regarding quality of life, we expect that a 15% difference in score between the groups is clinically significant.

5. Practical considerations and responsibilities (Norwegian part of study)

Patients are received at the obesity clinic at the first consultation at the hospital, and examined by a physician, research nurse and nutritionist.

The treating physician will clarify whether the patient is appropriate for the study and is responsible for informing the patient about the study, inviting for participation, and for obtaining signed informed consent. This doctor obtains appropriate blood tests, and is responsible for completing all forms including preoperative data.

The nutritionist will be responsible for informing and guiding the patient by filling in the questionnaires regarding quality of life and eating patterns. All participants receive standardized information about the postoperative diet.

Data regarding health economics are obtained from medical records (length of surgery, stay in recovery and intensive care, and total hospital stay). The responsible physician fills out these forms on the participant's departure.

At outpatient visits, data registration is performed by physician and nutritionist.

Time frame

Inclusion of participants will start approximately January 2006. All data will be recorded prospectively and consecutively. Analysis and publication of the short-term results – including hospital stay and 30-day complication rates – is planned for the first half of 2007.
Long-term results (2 years) are planned for publication later than the first half of 2009.
The study will involve 5 years of follow-up.

6. Ethical considerations

The study will be conducted in accordance with the Helsinki Declaration.

The study is to be approved by the regional ethics committees in both countries, and may only commence after approval is received. It is also applied for permission from The Data Inspectorate to register information on all patients treated for morbid obesity at OUH. The study has already been approved by the medical faculties at the Sahlgrenska Academy ethics committee, University of Gothenburg.

Both surgical methods have been established at the two hospitals, and are accepted methods for the treatment of super-obesity. There is no consensus on which surgical method that provides the best treatment outcome. This study aims to help clarify this and will provide new medical knowledge that is important. We see no ethical qualms about using both surgical methods.

Patients are not subjected to risks associated with additional tests or investigations solely carried out for study purposes.

Participation in the study is voluntary, and patients will be informed about the study both orally and in writing. Participants may withdraw from the study at any time without giving any reason for this.

The patients will be asked to participate in the study by the attending physician, and may as such experience some degree of pressure for participating. It must therefore be emphasized that participation in the study is voluntary.

All information obtained in the study will be treated confidentially and will only be known to the responsible employees.

7. Publications

We plan to present the results in scientific papers in international peer-reviewed journals with publications for each of the collaborators involved. This applies for Sofia Björkman, Torgeir T. Søvik, Osama Taha, and Sigrun Henjum. Erlend T. Aasheim already has a clear plan for his doctorate.

The research nurses may be offered co-authorships in studies regarding quality of life or in cases where they have been active in research in accordance with current regulations for authorship.

8. Funding (for the Norwegian part)

All employees have a salary at the respective departments. It will be applied for financial support from South-Eastern Norway Regional Health Authority.

9. Insurance

Standard patient insurance applies.

References

1. Gastrointestinal surgery for severe obesity. *Consens Statement* 1991;9:1-20.

2. Maggard MA, Shugarman LR, Suttorp M, et al. Meta-analysis: surgical treatment of obesity. *Ann Intern Med.* 2005;142:547-559.

3. Buchwald H, Avidor Y, Braunwald E, et al. Bariatric surgery: a systematic review and meta-analysis. *JAMA.* 2004;292:1724-1737.

4. Lonroth H. Laparoscopic gastric bypass. *Obes Surg.* 1998;8:563-565.

5. Brolin RE, Kenler HA, Gorman JH, Cody RP. Long-limb gastric bypass in the superobese. A prospective randomized study. *Ann Surg.* 1992;215:387-395.

6. Choban PS, Flancbaum L. The effect of Roux limb lengths on outcome after Roux-en-Y gastric bypass: a prospective, randomized clinical trial. *Obes Surg.* 2002;12:540-545.

7. Hess DS, Hess DW. Biliopancreatic diversion with a duodenal switch. *Obes Surg.* 1998;8:267-282.

8. Ren CJ, Patterson E, Gagner M. Early results of laparoscopic biliopancreatic diversion with duodenal switch: a case series of 40 consecutive patients. *Obes Surg.* 2000;10:514-523.
