# Supplementary material for: Ten-Year Outcomes Following Roux-en-Y Gastric Bypass vs Duodenal Switch for High Body Mass Index: A Randomized Clinical Trial
Source: JAMA Netw Open. 2024 Jun 3;7(6):e2414340. doi: 10.1001/jamanetworkopen.2024.14340 (PMC11148687; doi:10.1001/jamanetworkopen.2024.14340)
Supplement: Supplement 2. — eTable 1. Trial Definitions of Comorbidities and Their Resolutions eTable 2. Comorbidities and Vitamin Deficiencies 10 Years or More After Roux-en-Y Gastric Bypass (RYGB) and Duodenal Switch (DS) Surgery eTable 3. Adverse Events and Effects 30 Days After Surgery to 10 Years After Roux-en-Y Gastric Bypass (RYGB) and Duodenal Switch (DS) eTable 4. Vitamin and Mineral Supplementation Reported at Baseline and 5 and 10 Years After Roux-en-Y Gastric Bypass (RYGB) and Duodenal Switch (DS) Surgery eTable 5. Areal Bone Mineral Density (aBMD) for Patients 5 and 10 Years After Roux-en-Y Gastric Bypass (RYGB) and Duodenal Switch (DS) eTable 6. Serum Bone Turnover Markers 5 and 10 Years After Roux-en-Y Gastric Bypass (RYGB) and Duodenal Switch (DS) eTable 7. Gastrointestinal Symptoms Rating Scale (GSRS) eTable 8. Patient-Reported Experience Measures (PREM) 10 Years or More for 44 Patients After Roux-en-Y Gastric Bypass (RYGB [n = 21]) and Duodenal Switch (DS [n = 23]) Surgery eFigure 1. Areal Bone Mineral Density (aBMD) Scores From Dual Energy X-Ray Absorptiometry (DEXA) Scans for 60 Patients 5 and 10 Years After Roux-en-Y Gastric Bypass (n = 31) and Duodenal Switch (n = 29) Surgery eFigure 2. Short Form (SF-36) Questionnaire for 60 Patients at Baseline, 5 and 10 Years After Roux-en-Y Gastric Bypass (RYGB) (n = 31) and Duodenal Switch (DS) (n = 29) [file jamanetwopen-e2414340-s002.pdf]

Supplementary Online Content

Salte OBK, Olbers T, Risstad H, et al. Ten-year outcomes following Roux-en-Y gastric bypass vs duodenal switch for high body mass index: a randomized clinical trial. *JAMA Netw Open*. 2024;7(6):e2414340. doi:10.1001/jamanetworkopen.2024.14340

eTable 1. Trial Definitions of Comorbidities and Their Resolutions

eTable 2. Comorbidities and Vitamin Deficiencies 10 Years or More After Roux-en-Y Gastric Bypass (RYGB) and Duodenal Switch (DS) Surgery

eTable 3. Adverse Events and Effects 30 Days After Surgery to 10 Years After Roux-en-Y Gastric Bypass (RYGB) and Duodenal Switch (DS)

eTable 4. Vitamin and Mineral Supplementation Reported at Baseline and 5 and 10 Years After Roux-en-Y Gastric Bypass (RYGB) and Duodenal Switch (DS) Surgery

eTable 5. Areal Bone Mineral Density (aBMD) for Patients 5 and 10 Years After Roux-en-Y Gastric Bypass (RYGB) and Duodenal Switch (DS)

eTable 6. Serum Bone Turnover Markers 5 and 10 Years After Roux-en-Y Gastric Bypass (RYGB) and Duodenal Switch (DS)

eTable 7. Gastrointestinal Symptoms Rating Scale (GSRS)

eTable 8. Patient-Reported Experience Measures (PREM) 10 Years or More for 44 Patients After Roux-en-Y Gastric Bypass (RYGB [n = 21]) and Duodenal Switch (DS [n = 23]) Surgery

eFigure 1. Areal Bone Mineral Density (aBMD) Scores From Dual Energy X-Ray Absorptiometry (DEXA) Scans for 60 Patients 5 and 10 Years After Roux-en-Y Gastric Bypass (n = 31) and Duodenal Switch (n = 29) Surgery

eFigure 2. Short Form (SF-36) Questionnaire for 60 Patients at Baseline, 5 and 10 Years After Roux-en-Y Gastric Bypass (RYGB) (n = 31) and Duodenal Switch (DS) (n = 29)

This supplementary material has been provided by the authors to give readers additional information about their work.

© 2024 Salte OBK et al. *JAMA Network Open*.

eTable 1. Trial Definitions of Comorbidities and Their Resolutions

Type 2 diabetes:	o Fasting glucose ≥126 mg/dL(≥ 7.0 mmol/L)<br>o HbA1c ≥ 48 mmol/mol (≥ 6.5 %)<br>o And/or use of antidiabetic medications<br>o Absence of type 1 diabetes
Hypertension:	o Systolic BP ≥ 140 mmHg<br>o Diastolic BP ≥ 90 mmHg<br>o And/or use of antihypertensive drugs
Secondary hyperparathyroidism:	o PTH ≥ 66 pg/ml (≥ 7.0 pmol/L) *without* hypercalcemia<br>o Hypercalcemia: free/ionized calcium ≥ 5.16 mg/dL (≥ 1.29 mmol/L)
Iron deficiency:	o Ferritin ≤ 20 ng/ml (≤ 20 µg/L)<br>o Iron ≤ 50 g/µdL (≤ 9 µmol/L)<br>o And/or those with anemia
Anemia	o Hemoglobin ≤ 13.5 g/dL in men, ≤ 12.0 g/dL in women<br>o And/or need for iron infusion and/or blood transfusion
Dyslipidemia	o LDL cholesterol ≥ 116 mg/dL (≥ 3.0 mmol/L)<br>o HDL < 40 mg/dL (< 1.03 mmol/L) in men and < 50 mg/dL (< 1.29 mmol/L) in women<br>o Triglycerdies ≥ 150 mg/dL (≥ 1.7 mmol/L)<br>o Total cholesterol /HDL cholesterol ratio > 5.0<br>o And/or use of lipid lowering medications
Vitamin deficiencies	o Vitamin A < 10 µg/dL (< 0.35 µmol/L)(ref. 20-80 µg/dL)<br>o Vitamin B1 (Thiamine) <2.36 µg/dL (< 70 nmol/L) (ref 3.2-6.8 µg/dL)<br>o Vitamin B9 (Folate) < 3 ng/mL (<7 nmol/L)<br>o Vitamin B12 < 200 pg/mL (< 150 pmol/L)(ref 200-1000 pg/mL)<br>o Vitamin D (25 OH) < 20 ng/mL (< 50 nmol/L)(ref >30 ng/mL)<br>o And/or increased substitution therapy
Metabolic syndrome definition:<br>*(International Diabetes Federation)*	**Central obesity**<br>(waist circumference – European: ≥ 94 cm males, ≥ 80 cm females) or BMI ≥ 30 (then central obesity is assumed) + 2 or more of any of the following:<br>a) Triglycerides ≥ 150 mg/dL (≥ 1.7 mmol/l)<br>b) HDL cholesterol < 40 mg/dL (< 1.03 mmol/L) in males, < 50 mg/dL (< 1.29 mmol/L) in females<br>c) Raised blood pressure (≥ 130 mmHg systolic BP and/or ≥ 85 mmHg diastolic BP) or on antihypertensive medication<br>d) Fasting glucose ≥100 mg/dL (≥ 5.6 mmol/L) or diagnosis of type 2 diabetes
Osteoporosis	o DEXA: t-score ≤ -2.5 of lumbar spine, femoral neck and/or hip<br>And/or use of medication for osteoporosis
Osteopenia	o DEXA: t-score ≤ -1.0 to -2.4 of lumbar spine, femoral neck and/or hip
Resolution of Type 2 diabetes:	o HbA1c ≤ 42 mmol/mol (≤ 6.0 %)<br>o Fasting glucose < 100 mg/dL (≤ 5.55 mmol/L)<br>o And no use of antidiabetic medication
Partial resolution of Type 2 diabetes:	o HbA1c 39 - 46 mmol/mol (5.7 - 6.4 %)<br>o Fasting glucose 100 – 125 mg/dL (5.55 – 6.94 mmol/L)<br>And no use of antidiabetic medication
Resolution of hypertension	o Systolic BP < 140 mmHg<br>o And Diastolic BP < 90 mmHg<br>o And no use of antihypertensive medication

© 2024 Salte OBK et al. *JAMA Network Open*.

eTable 2. Comorbidities and Vitamin Deficiencies 10 Years or More After Roux-en-Y Gastric Bypass (RYGB) and Duodenal Switch (DS) Surgery

**Medical conditions, No (%)**	**Baseline***		**10 years after surgery****		**Difference between groups at 10 years**
	**RYGB (n= 31)**	**DS (n=29)**	**RYGB (n=23)**	**DS (n=25)**	**P-value**
Type 2 diabetes	5 (16)	6 (21)	2 (9)	1 (4)	.42
- Complete remission	N/A	N/A	3 (13)	4 (16)	.85
- Partial remission	N/A	N/A	2 (9)	0 (0)	.11
Hypertension	8 (26)	8 (28)	11 (48)	9 (36)	.47
Dyslipidemia	26 (84)	25 (86)	15 (65)	14 (56)	.89
OSAS/Sleep apnea	5 (16)	6 (21)	1 (4)	2(8)	.42
Metabolic syndrome	20 (65)	23 (79)	12 (52)	7 (28)	.22
Secondary hyperparathyroidism	N/A	N/A	7 (30)	15 (60)	.02
Cardiac disease	0 (0)	0 (0)	1 (4)	3 (12)	.23
Asthma	8 (26)	5 (17)	3 (13)	3 (12)	.84
Gastroesophageal reflux disease	5 (16)	4 (14)	4 (17)	10 (40)	.05
Gout	1 (3)	1 (3)	1 (4)	1 (4)	.75
Hypothyroidism	3 (10)	3 (10)	2 (9)	0 (0)	.36
Joint pain	16 (52)	13 (45)	13 (57)	16 (64)	.38
Depression	5 (16)	12 (41)	10 (43)	8 (32)	.68
Gallstones	2 (6)	1 (3)	7 (30)	13 (52)	.07
Ureteral stones	0 (0)	0 (0)	1 (4)	2 (8)	.42
Urinary incontinence	5 (16)	7 (24)	1 (4)	3 (12)	.23
Anemia	1 (3)	1 (3)	9 (39)	11 (44)	.50
Iron deficiency	1 (3)	1 (3)	11 (28)	14 (56)	.37
Vitamin A deficiency	0 (0)	0 (0)	0 (0)	4 (16)	.08
Vitamin B1 deficiency	0 (0)	1 (3)	1 (4)	2 (8)	.80
Vitamin B9 deficiency	0 (0)	0 (0)	3 (13)	4 (16)	.85
Vitamin B12 deficiency	0 (0)	0 (0)	4 (17)	1(4)	.12
Vitamin D(25-OH) deficiency	10 (33)	8 (29)	9 (39)	19 (76)	<.01
Vitamin deficiencies (any)	10 (33)	9 (31)	11 (48)	21 (84)	<.01

***Percentages calculated from baseline n-values **Percentages calculated from n-values 10 years after surgery

© 2024 Salte OBK et al. *JAMA Network Open*.

eTable 3. Adverse Events and Effects 30 Days After Surgery to 10 Years After Roux-en-Y Gastric Bypass (RYGB) and Duodenal Switch (DS)

**Adverse event, No (%)**	**RYGB (n=31)**	**DS (n=29)**	**P-value**
**Gastrointestinal**			
- Gallstones	7 (23)	13 (45)	.07
- Cholecystectomy	4 (13)	11 (38)	**.03**
- Elongation of common channel	0 (0)	3 (10)	**.04**
- Internal hernia	2 (6)	2 (7)	.95
- Bowel obstruction	2 (6)	2 (7)	.95
- Liver failure	0 (0)	1 (3)	.24
- Peptic ulcer	0 (0)	2 (7)	.12
- Barrett's esophagus	0 (0)	1 (3)	.24
- Diverticulitis	1 (3)	0 (0)	.74
- Appendicitis	2 (6)	0 (0)	.36
- Gastrointestinal bleeding	2 (6)	1 (3)	.80
- Blood transfusions	1 (3)	1 (3)	.75
- Abdominal pain	9	4 (14)	.15
**Nutritional**			
- Iron deficiency	11 (35)	14 (48)	.31
- Anemia	9 (29)	11 (38)	.47
- Iron injections	4 (13)	6 (21)	.40
- Night blindness due to vitamin A deficiency	0 (0)	1 (3)	.24
- Protein-caloric malnutrition	0 (0)	4 (14)	**.02**
**Infections**			
- Any infection	5 (16)	10 (34)	.10
- Pneumonia	1 (3)	0 (0)	.74
- Urinary tract infections	0 (0)	4 (14)	**.02**
- Other infections	4 (13)	6 (21)	.40
**Other adverse events**			
- Ureteral stones	1 (3)	3 (10)	.23
- Renal failure	0 (0)	2 (7)	.11
- Arthritis	0 (0)	1 (3)	.24
- Arthrosis	3 (10)	1 (3)	.48
- Hypoglycemia	0 (0)	1 (3)	.24
- Osteoporosis	4 (17)	10 (34)	.15
- Osteopenia	7 (23)	11 (38)	.54
- Fractures	3 (10)	4 (14)	.56
- Severe psychiatric illness	3 (10)	3 (10)	.84
- Depression	9 (29)	8 (28)	.54
- Alcohol abuse	1 (3)	0 (0)	.74
- Type 1 diabetes	1 (3)	0 (0)	.74
- Cancer	1 (3)	0 (0)	.74
- Thromboembolic event	2 (6)	0 (0)	.36
- Inguinal hernia	0 (0)	1 (3)	.24
- Umbilical hernia	1 (3)	0 (0)	.74
- Ventral hernia	1 (3)	0 (0)	.74
**Deceased** *	2 (6)	1 (3)	.80
**Total number of adverse events**	97	135	**.02**
Number (%) of patients with adverse events	27 (87)	27 (93)	.44
Mean (SD) number of adverse events per patient	3.1 (2.5)	4.6 (2.6)	**.01**
Number of revisional surgeries (%)	0 (0)	3 (10)	**.05**
Number of hospital admissions	53	51	.82
Number of patients with hospital admissions (%)	21 (68)	18 (62)	.65
Number of patients with abdominal surgical procedures (%)	10 (32)	14 (48)	.20
Number of abdominal surgical procedures	11	18	.17
Number of patients with plastic surgical procedures (%)	7 (23)	11 (38)	.20
Number of plastic surgical procedures	12	17	.29

* Causes of death were severe protein-caloric malnutrition 13 years after DS (common channel elongated three years after DS). Two patients died 4 and 5 years after RYGB respectively, due to cancer

© 2024 Salte OBK et al. *JAMA Network Open*.

eTable 4. Vitamin and Mineral Supplementation Reported at Baseline and 5 and 10 Years After Roux-en-Y Gastric Bypass (RYGB) and Duodenal Switch (DS) Surgery

	**RYGB,** No. Patients (%)			**DS,** No. Patients (%)		
	**Baseline (n=31)**	**5 Years (n=27)**	**10 years (n=23)**	**Baseline (n=29)**	**5 Years (n=28)**	**10 years (n=25)**
Multivitamin/mineral	4 (13)	13 (48)	15 (65)	2 (7)	15 (54)	19 (76)
Calcium/vitamin D	1 (3)	10 (37)	18 (78)	0 (0)	15 (54)	19 (76)
Vitamin B12[a]	1 (3)	18 (67)	20 (87)	0 (0)	10 (36)	17 (68)
Iron	0 (0)	7 (26)	16 (70)	0 (0)	11 (39)	17 (68)
Added supplements[b]	1 (3)	6 (22)	9 (39)	1 (3)	9 (32)	15 (60)
Added vitamin-D[c]	0	1 (4)	5 (22)	0 (0)	6 (21)	9 (36)

Patients who reported using a supplement ≥5 days a week were considered as users of that supplement.
[a]Only gastric bypass patients were routinely recommended regular supplementation with vitamin B12, duodenal switch patients were recommended B12 supplementation only if levels were below reference values.
[b]Included additional supplementation of vitamins and minerals.
[c]Vitamin-D supplementation above recommended doses

© 2024 Salte OBK et al. *JAMA Network Open*.

eTable 5. Areal Bone Mineral Density (aBMD) for Patients 5 and 10 Years After Roux-en-Y Gastric Bypass (RYGB) and Duodenal Switch (DS)

	**5 years** mean (95% CI) (RYGB n=23, DS n=28)	**Between group difference at 5 years** mean (95% CI); P-value	**10 years** Mean (95% CI) (RYGB n=20, DS n=25)	**Between group difference at 10 years** mean (95% CI); P-value
**aBMD (total),** g/cm^2				
RYGB	1.20 (1.08 to 1.32)	0.13 (0.07 to 0.20); P<.01	1.21 (0.88 to 1.42)	0.17 (0.07 to 0.26); P< .01
DS	1.12 (1.08 to 1.17)		1.04 (0.74 to 1.43)	
**t-score (total)**				
RYGB	1.4 (-1.0 to 3.4)	N/A	1.1 (-1.8 to 3.4)	N/A
DS	-0.1 (-2.7 to 2.1)		-0.5 (-3.4 to 2.5)	
**z-score (total)**				
RYGB	0.1 (-1.9 to 1.9)	N/A	0.1 (-2.2 to 2.9)	N/A
DS	-1.1 (-3.3 to 1.1)		-1.3 (-3.9 to 1.5)	
**aBMD (L_1-L_4),** g/cm^2				
RYGB	1.19 (0.86 to 1.43)	0.06 (-0.08 to 0.20); P=.37	1.17 (0.87 to 1.47)	0.12 (0.02 to 0.23); P=.02
DS	1.13 (0.84 to 1.37)		1.04 (0.66 to 1.50)	
**t-score (L_1-L_4)**				
RYGB	0.0 (-2.7 to 2.2)	N/A	-0.2 (-2.8 to 2.4)	N/A
DS	-0.6 (-3.3 to 1.4)		-1.2 (-4.3 to 2.1)	
**z-score (L_1-L_4)**				
RYGB	-0.9 (-3.5 to 1.2)	N/A	-0.9 (-3.4 to 1.4)	N/A
DS	-1.3 (-3.8 to 1.4)		-1.8 (-4.5 to 1.6)	
**aBMD (femoral neck),** g/cm^2				
RYGB	1.00 (0.76 to 1.32)	0.09 (-0.02 to 0.20); P=.10	0.98 (0.73 to 1.19)	0.13 (0.05 to 0.21); P<.01
DS	0.91 (0.73 to 1.05)		0.85 (0.66 to 1.03)	
**t-score (femoral neck)**				
RYGB	0.0 (-1.8 to 2.3)	N/A	-0.4 (-2.2 to 1.7)	N/A
DS	-0.8 (-2.1 to 0.6)		-1.3 (-2.7 to 0.4)	
**z-score (femoral neck)**				
RYGB	-0.4 (-2.2 to 1.7)	N/A	-0.4 (-1.9 to 1.3)	N/A
DS	-1.1 (-2.3 to -0.1)		-1.4 (-3.1 to 0.8)	
**aBMD (femur total),** g/cm^2				
RYGB	1.07 (0.83 to 1.33)	0.13 (0.04 to 0.23); P<.01	1.04 (0.72 to 1.35)	0.18 (0.09 to 0.27); P<.01
DS	0.94 (0.83 to 1.11)		0.86 (0.53 to 1.17)	
**t-score (femur total)**				
RYGB	0.5 (-1.4 to 2.7)	N/A	0.2 (-2.3 to 2.9)	N/A
DS	-0.8 (-1.8 to 1.0)		-1.2 (-3.9 to 1.3)	
**z-score (femur total)**				
RYGB	-0.1 (-1.7 to 1.9)	N/A	-0.1 (-2.2 to 2.2)	N/A
DS	-1.2 (-2.0 to 0.2)		-1.6 (-3.2 to 0.4)	

© 2024 Salte OBK et al. *JAMA Network Open*.

eTable 6. Serum Bone Turnover Markers 5 and 10 Years After Roux-en-Y Gastric Bypass (RYGB) and Duodenal Switch (DS)

	**5 years** mean (95% CI) RYGB n=27 DS n=28	**Between group difference at 5 years** Mean (95% CI); p-value	**10 years** mean (95% CI) RYGB n=21 DS n=20	**Between group difference at 10 years** Mean (95% CI); p-value
**CTX-1**, ug/L				
RYGB	0.57 (0.43 to 0.71)	-0.25 (-0.48 to -0.02); P=.04	0.52 (0.41 to 0.63)	-0.19 (-0.44 to -0.06); P=.13
DS	0.82 (0.63 to 1.01)		0.71 (0.44 to 0.98)	
**P1NP**, ug/L				
RYGB	59.0 (50.7 to 67.3)	-47.7 (-70.3 to -25.0); P<.01	67.5 (54.5 to 80.6)	-49.5 (-102.5 to 3.6); P=.07
DS	106.7 (128.1 to 101.7)		125.8 (62.2 to 189.5)	
**BALP**, ug/L				
RYGB	39.1 (32.5 to 45.7)	-13.2 (-24.6 to -1.9); P=.02	43.8 (34.4 to 53.2)	-36.7 (-70.3 to -3.1); P=.03
DS	52.4 (42.9 to 81.8)		80.5 (47.9 to 113.1)	

Abbreviations, Carboxyl terminal telopeptide of type 1 collagen (CTX-1), Procollagen type 1 N-terminal propeptide (P1NP), Bone specific alkaline phosphatase (BALP). Parathyroid hormone (PTH), Reference ranges: *CTX-1 µg/L*: females 25-49 years: ≤ 0.57, ≥50 years: ≤1.01, males 30-50 years: ≤ 0.58 , 51-70 years: ≤ 0.7; *P1NP µg/L*: females >25 years: 11-94, males >25 years: 20-91; *BALP µg/L:* 5.5-24.6; PTH pg/mL: 14.1-66.0; Free calcium mg/dL: 4.60-5.32 (1.15-1.33 *mmol/L)*

© 2024 Salte OBK et al. *JAMA Network Open*.

eTable 7. Gastrointestinal Symptoms Rating Scale (GSRS)

A. 60 patients at baseline, 5 and 10 years after Roux-en-Y gastric bypass (RYGB) and duodenal switch (DS).

	**Baseline/preop** mean (95% CI) RYGB n=31 DS n=29	**5 years** mean (95% CI) RYGB n=27 DS n=27	**10 years** mean (95% CI) RYGB n=22 DS n=22	**Difference between groups at 10 years** mean (95% CI); P-value
**Reflux score**				
RYGB	1.5 (1.3 to 1.8)	1.1 (0.9 to 1.3)	1.3 (1.0 to 1.7)	-1.2 (-2.0 to -0.3); P=.01
DS	1.7 (1.3 to 2.0)	2.0 (1.4 to 2.6)	2.6 (1.7 to 3.4)	
**Abdominal pain score**				
RYGB	1.8 (1.5 to 2.0)	2.7 (2.2 to 3.2)	2.8 (2.2 to 3.4)	0.1 (-0.7 to 0.8); P=.89
DS	1.9 (1.6 to 2.2)	2.5 (2.1 to 3.0)	2.6 (2.1 to 3.2)	
**Indigestion score**				
RYGB	2.2 (1.8 to 2.5)	3.0 (2.4 to 3.5)	3.4 (2.8 to 4.0)	0.2 (-0.7 to 1.0); P=.70
DS	2.2 (1.8 to 2.6)	3.4 (2.9 to 3.9)	3.1 (2.5 to 3.7)	
**Diarrhea score**				
RYGB	1.8 (1.5 to 2.2)	2.3 (1.8 to 2.8)	2.7 (2.0 to 3.4)	-0.8 (-1.8 to 0.1); P=.08
DS	1.9 (1.6 to 2.3)	3.0 (2.4 to 3.6)	3.6 (2.9 to 4.2)	
**Constipation score**				
RYGB	1.7 (1.3 to 2.1)	1.8 (1.4 to 2.1)	2.2 (1.7 to 2.7)	0.2 (-0.4 to 0.8); P=.47
DS	1.5 (1.3 to 1.8)	1.7 (1.4 to 1.9)	1.9 (1.5 to 2.4)	

Each score has a minimum of 1 point (no complaint) and a maximum of 7 points (maximal complaint) possible

Bothersome symptoms are defined as having a score of 3 or more of any given score at the individual level

© 2024 Salte OBK et al. *JAMA Network Open*.

B. Bothersome symptoms 10 years after surgery for 44 patients after Roux-en-Y gastric bypass (RYGB) (n=22) and duodenal switch DS (n=22).

	Score < 3	Score 3 or more (Bothersome)	P-value
**Reflux**			
RYGB	19	3	.04
DS	13	9	
**Abdominal pain**			
RYGB	11	11	.38
DS	8	14	
**Indigestion**			
RYGB	5	17	.22
DS	9	13	
**Diarrhea**			
RYGB	12	10	.02
DS	4	18	
**Constipation**			
RYGB	13	9	.10
DS	18	4	

© 2024 Salte OBK et al. *JAMA Network Open*.

eTable 8. Patient-Reported Experience Measures (PREM) 10 Years or More for 44 Patients After Roux-en-Y Gastric Bypass (RYGB [n = 21]) and Duodenal Switch (DS [n = 23]) Surgery

	RYGB (n=21)	DS (n=23)	P-value
A) Are you experiencing adverse effects or complications after your surgery?[1]	3.9	4.1	.87
B) How pleased are you with the result of your surgery, all things considered?[2]	7.1	7.1	.97
C) Would you have recommended the surgery to others in your situation?[3]	8.3	8.3	.78

Each question graded freely from 0-10 points on a visual scale of 10 cm along a line which was measured and scored. Mean values are presented.

1. 0 refers to: 'Never' and 10: 'Very often'.
2. 0 refers to: 'Very unsatisfied' and 10: 'Very satisfied'.
3. 0 refers to: 'No, never' and 10: 'Yes, absolutely'.

© 2024 Salte OBK et al. *JAMA Network Open*.

eFigure 1. Areal Bone Mineral Density (aBMD) Scores From Dual Energy X-Ray Absorptiometry (DEXA) Scans for 60 Patients 5 and 10 Years After Roux-en-Y Gastric Bypass (n = 31) and Duodenal Switch (n = 29) Surgery

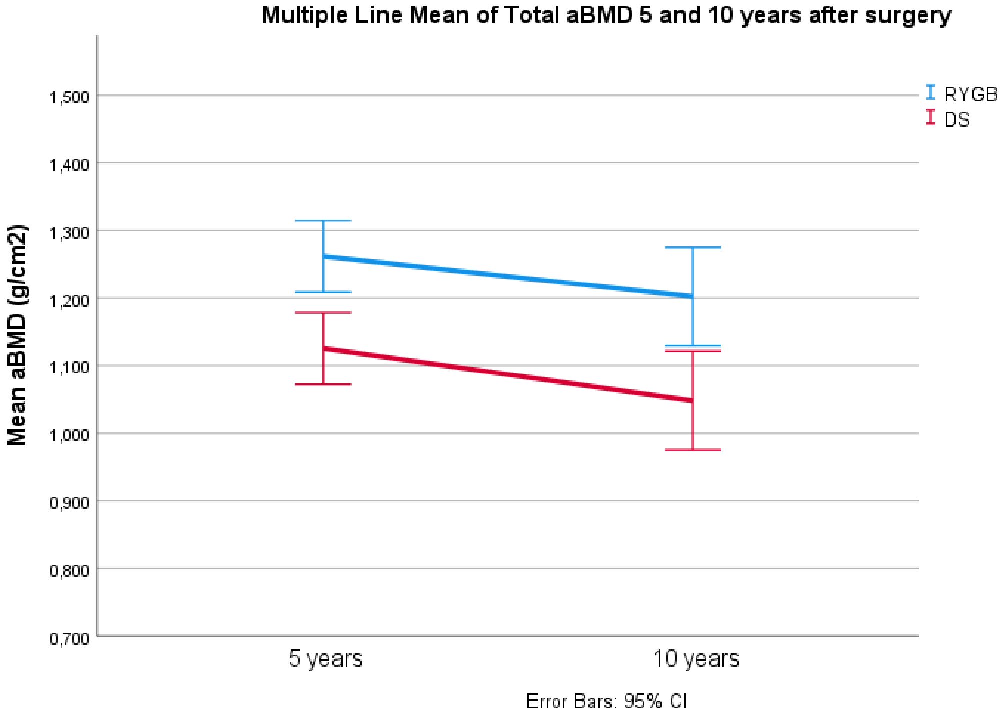

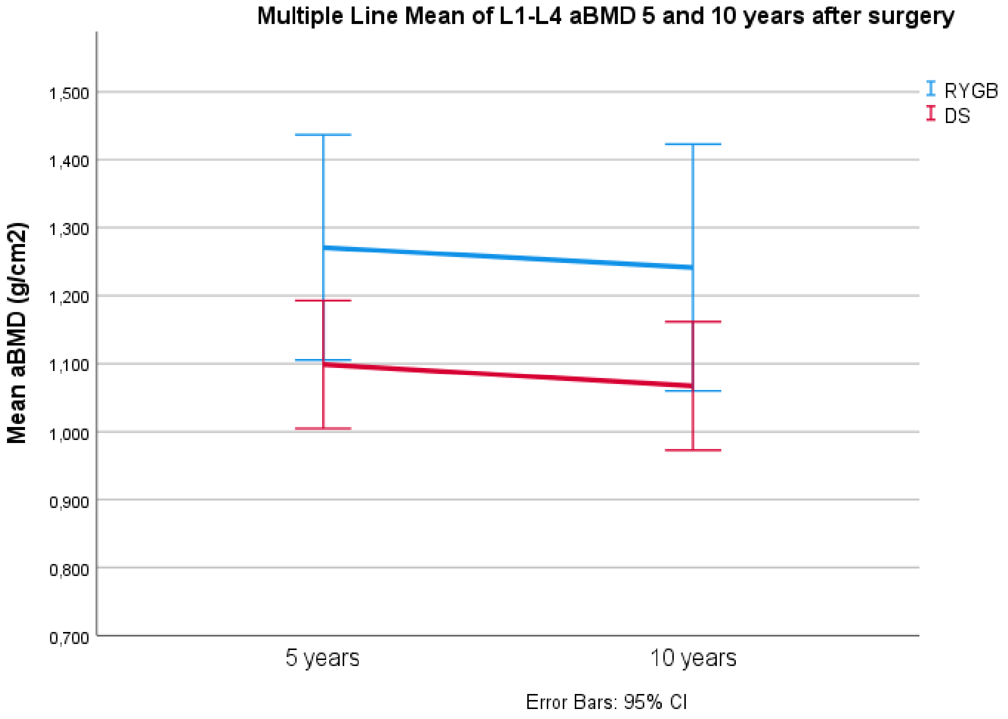

© 2024 Salte OBK et al. *JAMA Network Open*.

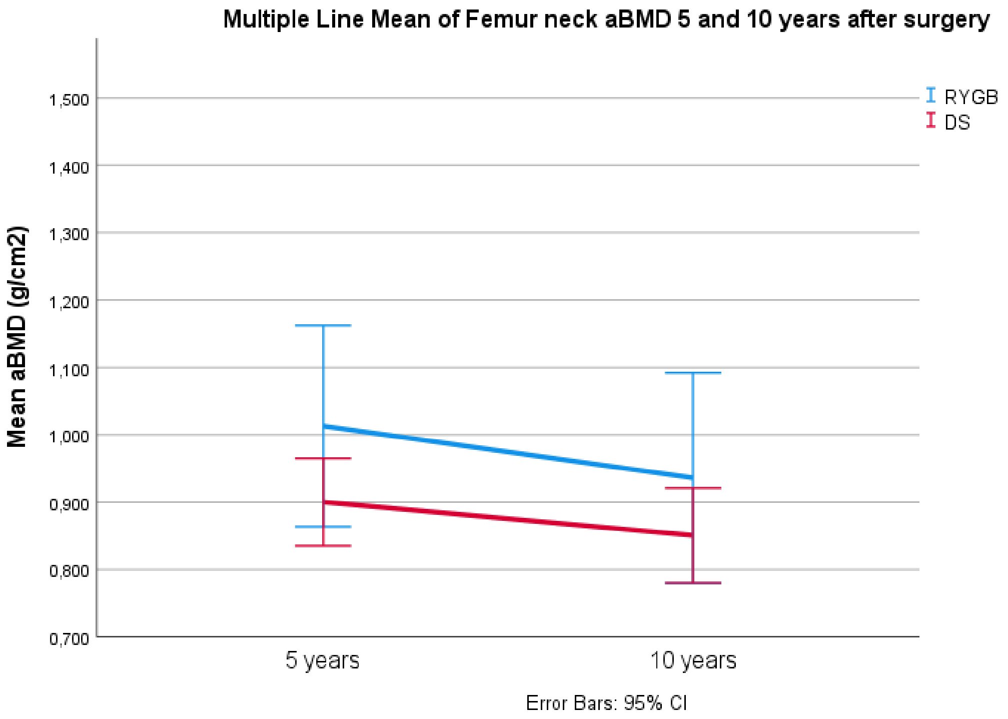

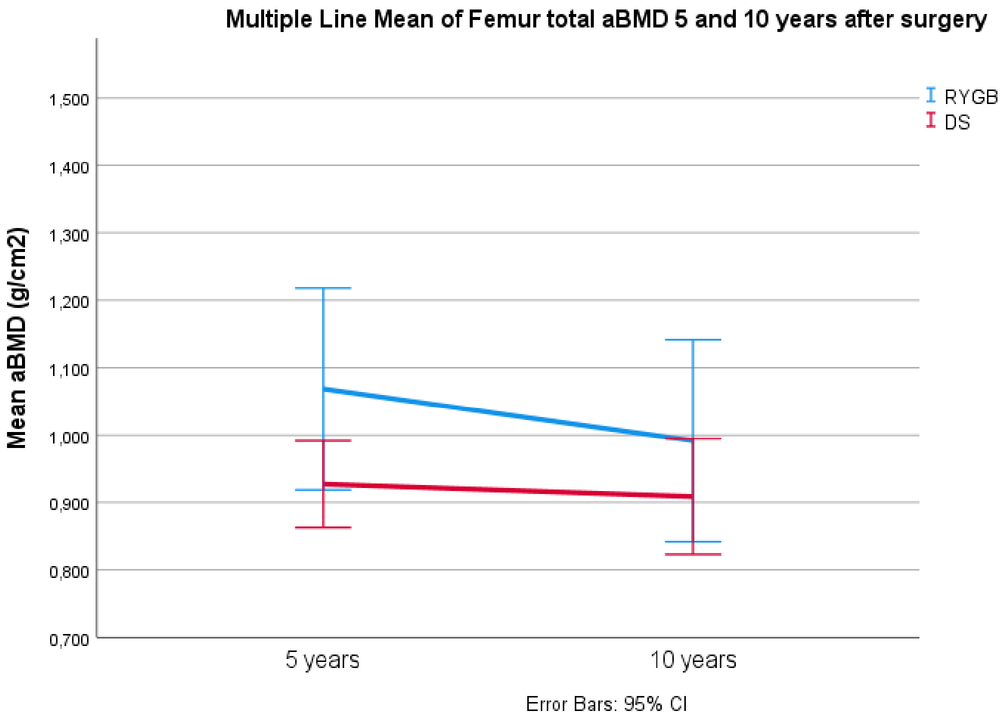

© 2024 Salte OBK et al. *JAMA Network Open*.

eFigure 2. Short Form (SF-36) Questionnaire for 60 Patients at Baseline, 5 and 10 Years After Roux-en-Y Gastric Bypass (RYGB) (n = 31) and Duodenal Switch (DS) (n = 29)

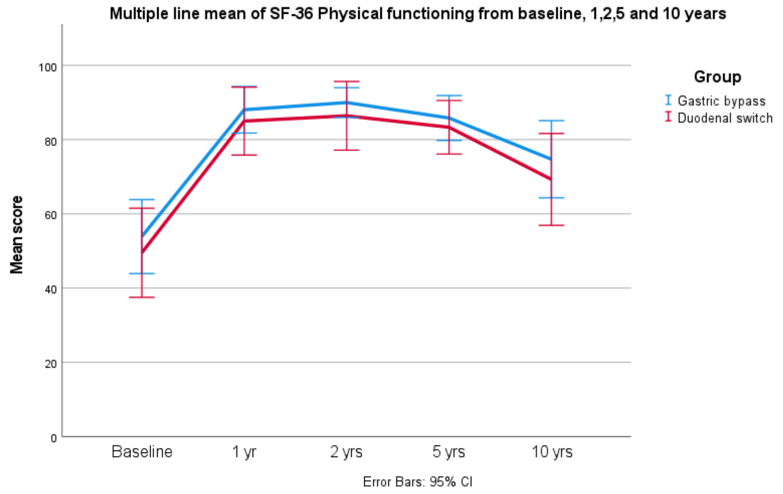

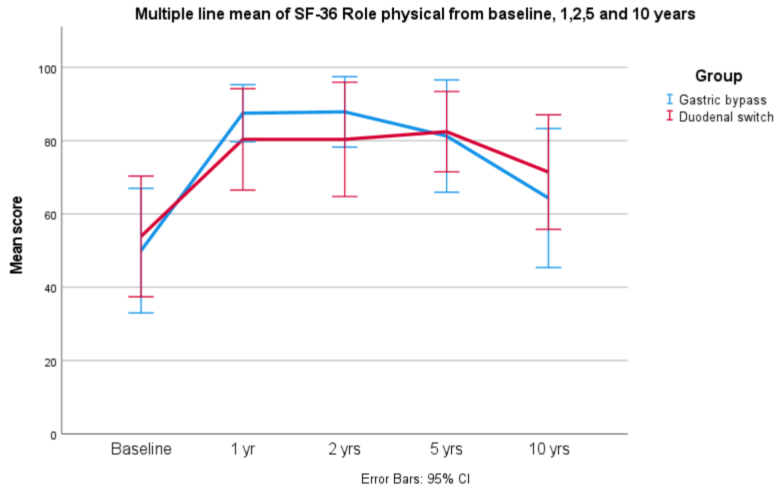

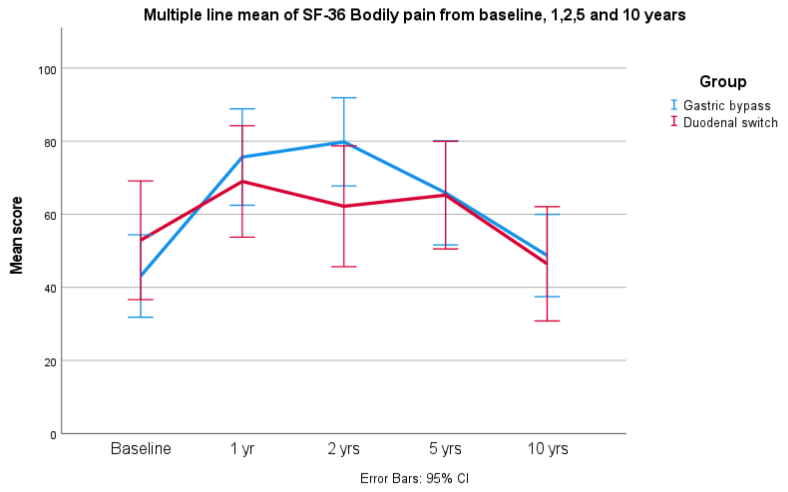

© 2024 Salte OBK et al. *JAMA Network Open*.

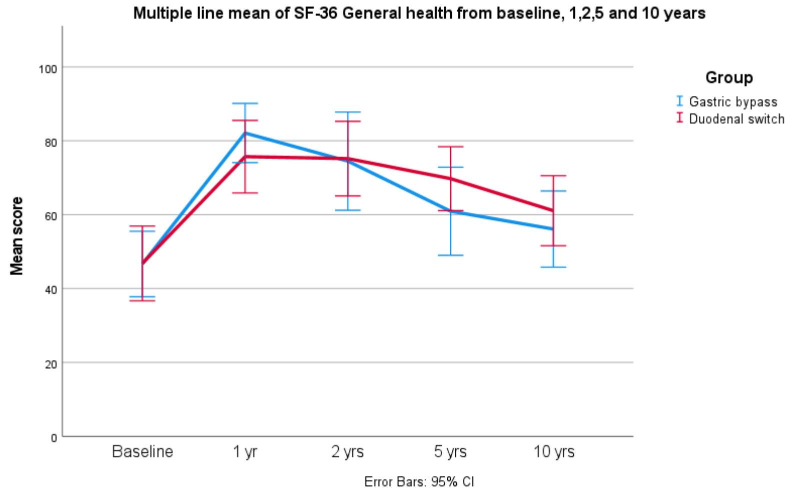

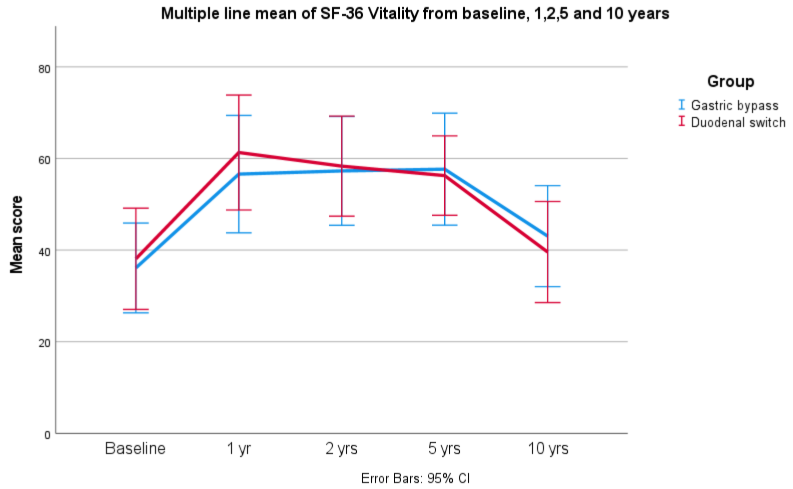

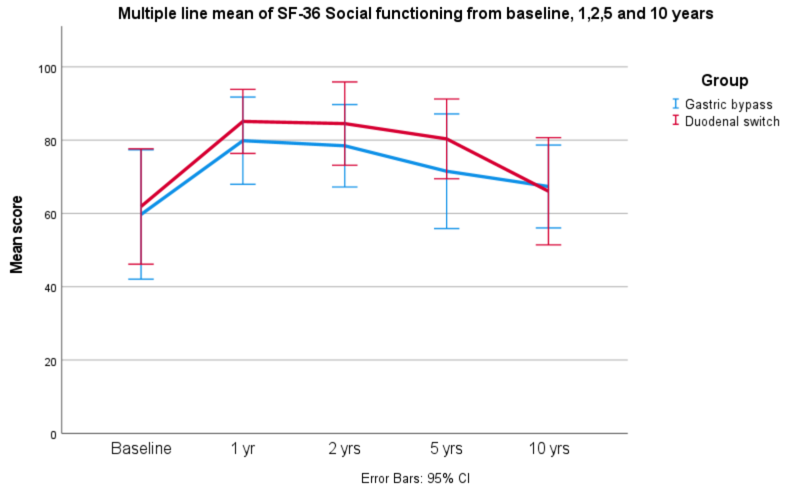

© 2024 Salte OBK et al. *JAMA Network Open*.

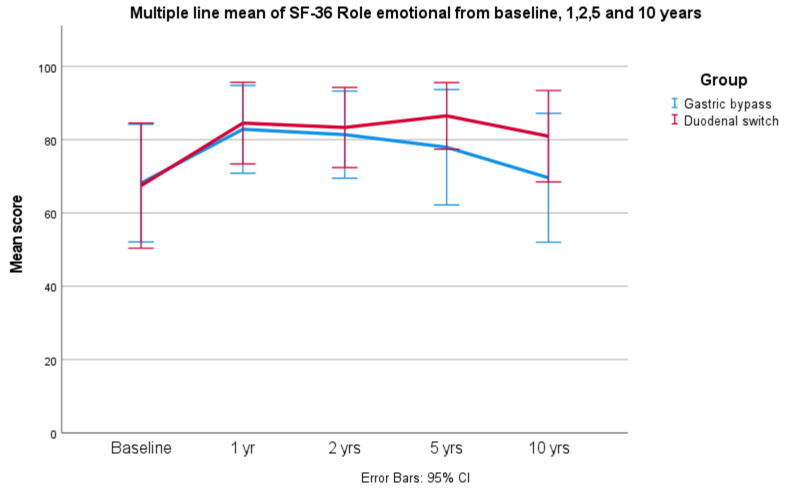

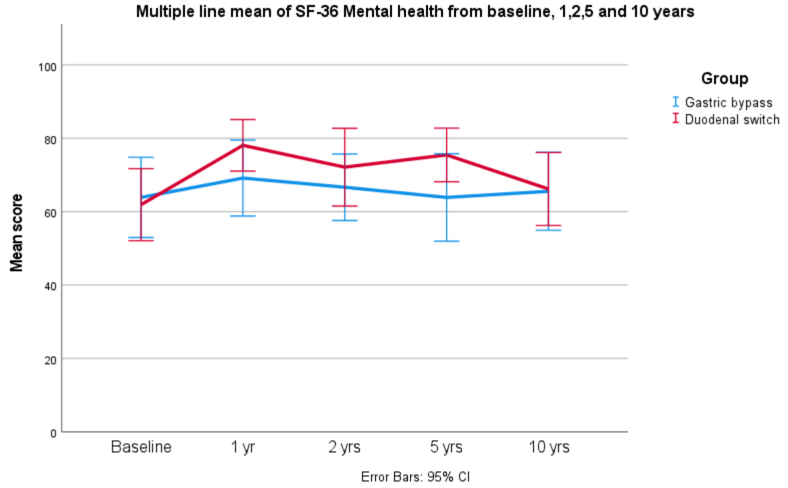

Interpretation of mean scores (0-100): 100 = best possible health score, 0 = worst possible health score

© 2024 Salte OBK et al. *JAMA Network Open*.
