# Supplementary material for: Ten-Year Outcomes Following Roux-en-Y Gastric Bypass vs Duodenal Switch for High Body Mass Index: A Randomized Clinical Trial
Source: JAMA Netw Open. 2024 Jun 3;7(6):e2414340. doi: 10.1001/jamanetworkopen.2024.14340 (PMC11148687; doi:10.1001/jamanetworkopen.2024.14340)
Supplement: Supplement 3. — Data Sharing Statement [file jamanetwopen-e2414340-s003.pdf]

Data Sharing Statement

Salte. Ten-Year Outcomes Following Roux-en-Y Gastric Bypass vs Duodenal Switch for High Body Mass Index. *JAMA Netw Open*. Published June 03, 2024.
doi:10.1001/jamanetworkopen.2024.14340

Data
Data available: Yes
Data types: Anonymized data
How to access data: oddsal@ous-hf.no
When available: With publication

Supporting Documents
Document types: None

Additional Information
Who can access the data: Anyone requesting the data
Types of analyses: For any purpose
Mechanisms of data availability: With investigator support
